# The Role of Valence and Uniqueness of Emotions in the Context of Infrahumanization Theory

**DOI:** 10.11621/pir.2022.0111

**Published:** 2022-03-30

**Authors:** Maria A. Terskova, Elena R. Agadullina

**Affiliations:** a HSE University, Moscow, Russia

**Keywords:** Infrahumanization, emotion, valence, uniqueness

## Abstract

**Background:**

Infrahumanization is a result of group comparison when the ingroup is considered as fully human in comparison to an outgroup that is viewed as lacking humanness and similar to animals. Infrahumanization theory proposed that the attribution of emotions to ingroups and out-groups is based on their uniqueness, regardless of the valence of these emotions. Since the valence of information plays an important role in its processing and perception, it was decided to clarify the role of uniqueness and valence.

**Objective:**

This article aims to explore the role of valence and uniqueness in the perception of emotions within the framework of the infrahumanization theory.

**Design:**

Three studies were conducted. A preliminary study selected emotions with extreme values for uniqueness and valence to create a list for measuring infrahumanization for the Russian socio-cultural context. In Study 1, we tested three alternative models of perception of emotions’ uniqueness and valence. In Study 2, we replicate the results from Study 1 and check the robustness of the models obtained.

**Results:**

In a preliminary study (N = 146), twelve emotions with different levels of uniqueness and valence were selected for the Russian sociocultural context. CFA was used for testing the models in Studies 1 and 2. The results of Study 1 (N = 243) demonstrated the role of valence and uniqueness in the perception of emotions. Study 2 (N = 482) confirmed the results obtained in Study 1.

**Conclusion:**

For the first time, the infrahumanization measure was adapted to the Russian socio-cultural context. Infrahumanization research should control valence for a qualitative discussion of the results.

## Introduction

At the turn of the century, Leyens et al. (2001; 2000) described the infrahumanization theory, which proposed a new approach to understanding intergroup relationships, based on the idea of the humanity of different groups. According to Leyens et al., people believe that there is a unique ‘human essence’, which includes intelligence, language, and sentiments (secondary emotions). Within the framework of the infrahumanization theory, Leyens et al., focused on the analysis of sentiments. They claimed that in contrast to primary emotions (e.g., joy, surprise, fear, anger), which humans share with other animals, secondary emotions (e.g., love, hope, contempt, resentment) are unique to humans. The process through which secondary emotions (regardless of their valence) are attributed to an ingroup more than to an outgroup, and the absence of such differences for primary emotions was called infrahumanization.

Infrahumanization is a result of group comparison that links with positive ingroup bias when the ingroup is seen as fully human in comparison to an outgroup that is viewed as lacking humanness. The main difference between infrahumanization and ingroup favoritism is that infrahumanization is based on the attribution of both positive and negative secondary emotions to groups, since valence does not itself make these emotions more or less human. According to Haslam and Loughnan (2014), the concept ‘infrahumanization’ makes a significant theoretical advance in intergroup studies because it changes the way ingroup bias is viewed, highlighting the fact that it can be based on the attribution of characteristics regardless of their valence.

Over the 20 years since the infrahumanization theory was formulated, the infrahumanization effect has been studied on ethnic groups (Bain et al., 2009), national groups (Davies et al., 2018), gender groups (Viki & Abrams, 2003), age groups (Boudjemadi et al., 2017), religious group (Enock et al., 2021), and professional groups (Iatridis, 2013). The researchers also analyzed the consequences of the infrahumanization effect on intergroup relationships. In particular, it was found that infrahumanization reduces empathy with outgroup victims (Castano & Giner-Sorolla, 2006), increases the perception of the outgroup as threatening the values of the ingroup (Pereira et al., 2009), and is associated with the approval of violence against the outgroup (Motyl et al., 2010).

But in recent years, questions have accumulated regarding the infrahumanization theory, one of which is associated with the value of the emotions’ valence in their perception and attribution.

Scientists have widely described the role of the valence of information in its perception, memorization, and reproduction (for example, the positive-negative asymmetry effect; Baumeister et al., 2001). Today, it is well known that, under certain conditions, positive or negative information can have a large impact on perception. In particular, negative information is more noticeable and memorable since it is, on average, more distinguishable from other negative information. This is why negative emotions are detected faster (Hansen & Hansen, 1988). At the same time, according to the density hypothesis, positive information is, on average, more similar to other positive information and this leads to stronger impact on the formation of impressions (Gräf & Unkelbach, 2016).

Even though the infrahumanization theory suggests that the valence of emotions is not important in attributing secondary emotions, Leyens et al. (2001) recognized the impact of valence in the attribution of primary emotions. The authors emphasized that it is possible that more positive primary emotions would be attributed to an ingroup than to an outgroup because of the ingroup favoritism effect. Over time, a lot of data has accumulated that, in one way or another, demonstrate the role of emotions’ valence in attributing the emotions to groups. Researchers began to formulate questions about the role of valence in infrahumanization theory and to assume the parallel occurrence of the effects associated with the valence and uniqueness of emotions (see, for example, Eyssel & Ribas, 2012).

Three patterns can be distinguished from the infrahumanization studies. The first pattern corresponds to the ingroup favoritism and outgroup hostility effects when more positive emotions are attributed to an ingroup and negative emotions to an out-group. Gaunt (2009) found that Jews (ingroup) attribute more positive emotions to themselves than to Arabs (outgroup) and more negative emotions to outgroup than to the ingroup. Iatridis (2013), in a study of professions with lawyers as an ingroup and shopkeepers as an outgroup, found that the former tend to attribute more secondary and positive emotions to the ingroup than to the outgroup.

In another group of studies, the second pattern—only positive asymmetry—was observed (in general, more positive emotions are attributed regardless of their type). For example, such a result was obtained in Australian and Chinese samples (Bain et al., 2009). Finally, there are studies with the third pattern in which more negative emotions are attributed in general (only negative asymmetry). This effect is observed in the example of lawyers (ingroup) and shopkeepers (outgroup; Iatridis 2013).

Importantly, in many studies it is impossible to draw a conclusion about the role of valence in the attribution of emotions since the authors do not describe the results of such an analysis. Instead, they state that there should be no differences in the attribution of secondary emotions of different valences (Albarello & Rubini, 2012), or they use emotions with only one valence (e.g., Demoulin et al., 2009).

As a result, the question about the role of emotions’ valence in their perception and attribution remains open. The main research question of this paper is: ’What role does valence and the perceived uniqueness of emotions play in their perception and attribution?’ A well-grounded answer to this question is important for a correct understanding and interpretation of the results obtained in the framework of the infrahumanization theory, as well as for the development of the theoretical assumptions of this theory.

## Overview of the studies

The main goal of this paper is to study the role of valence and uniqueness in the perception of emotions. To achieve this goal, three studies were planned and carried out. In a preliminary study (N = 146), we selected emotions with different valence that are perceived as primary and secondary in the Russian socio-cultural context. In Study 1 (N = 243) we tested three alternative models of the perception of emotions’ uniqueness and valence. The first model assumed that uniqueness is the dominant feature in the perception of emotions and, as a result, two factors are formed: primary and secondary emotions. The second model suggested that valence is the dominant feature in the perception of emotions and two factors are formed: positive and negative emotions. The third model (bifactor) assumed that both attributes (uniqueness and valence) could play an important role in perception simultaneously. In Study 2 (N = 482) we re-tested all three models to check the robustness of the results obtained in Study 1.

## Preliminary study

One of the features of measuring infrahumanization is that cultural differences may exist in the perception of emotions as primary or secondary. For example, Bain et al. (2012) demonstrated that respondents from individualistic and collectivistic cultures differently perceive emotions as reflecting human essence and uniqueness. It is difficult to isolate patterns of infrahumanization effect across cultures: some studies do not report all comparisons and effects. However, there is a fair amount of research with culture-specific lists of primary and secondary emotions that has been conducted in more individualistic (France and Germany; Boudjemadi et al., 2017; Eyssel & Ribas, 2012), and more collectivistic (Greece and Portugal; Iatridis, 2013; Vala et al., 2009) cultures. There have also been examples that are a blend of individualistic and collectivist cultures (Hofstede et al., 2010): Israel (Gaunt, 2009), Spain (Rodríguez et al., 2016).

Russian culture cannot be unambiguously classified as individualistic or collectivistic (Naumov & Puffer, 2000), which is why the lists of emotions proposed in previous studies may not fully fit the Russian context. Previously, no infrahumanization research has been conducted in Russia and there is no measurement tool. The preliminary study aimed to determine the list of uniquely (secondary) and non-uniquely (primary) emotions that are relevant for the Russian socio-cultural context.

In return for a course credit, 54 students from a large Moscow university (66.7 % women, *M_age_* = 17.85, *SD* = .66) rated 146 emotions which were identified in a previous study (Liusin & Sinkevich, 2010). Respondents categorized each emotion from 1 to 3 (1 — this emotion is typical both for humans and animals; 2 — this emotion is unique to humans; 3 — difficult to answer). The emotion was considered primary or secondary and positive or negative when at least 80 percent of the respondents categorized it into a certain group. On this stage, we selected 40 out of 146 emotions based on their categorization as uniquely and non-uniquely human.

At the next stage, 92 respondents (53.0 % women, *M_age_* = 32.12, *SD* = 3.04) rated each emotion on a 10-point scale of uniqueness (from 1 = *is not unique to humans: both humans and other living things are able to experience this emotion,* to 10 = *is unique to humans: only humans are able to experience this emotion*) and valence (from 1 = *it is a negative emotion* to 10 = *it is a positive emotion*).

For the next studies, we selected emotions that met the following criteria: the emotion was evaluated as high (*M* + *SD*) or low (*M* – *SD*) on the uniqueness item (*M_unique_* = 5.96, *SD* = 1.98; upper thresholds for uniqueness — 7.94, lower – 3.98) and the same emotion was evaluated as high or low on the valence item (*M_valence_* = 5.18, *SD* = 2.14; upper thresholds for valence — 7.32, lower — 3.04).

We selected twelve emotions with different valence and perceived uniqueness that met the criteria: three primary positive emotions (joy, *радость*; pleasure, *удовольствие*; calmness, *спокойствие*), three primary negative emotions (pain, *боль*; anger, *злость*; fear, *страх*), three secondary positive emotions (inspiration, *вдохновение*; enthusiasm, *воодушевление*; admiration, *восхищение*), and three secondary negative emotions (gloating, *злорадство*; pessimism, *пессимизм*; emptiness, *опустошенность*)^1^.

The highlighted list of primary positive and negative emotions generally coincides with those emotions that are used in other countries (e.g., Greece, Portugal, Israel, and Spain). This result may indicate a cultural universality in the perception of primary emotions. The main differences are related to secondary emotions, especially negative ones. These differences can be associated with the fact that in individualistic cultures, emotions have a greater intrapersonal meaning, confirming the importance of the individual (Suh et al., 1998), while in collectivistic cultures, emotions are important in the context of groups and interpersonal relationships. The list of secondary emotions we have obtained is mostly different from those emotions that were previously used in various cultural contexts, which once again emphasizes the importance of the preliminary selection of emotions that are relevant to a particular culture. Thus, for the first time, we have identified a list of primary and secondary emotions specific to the Russian socio-cultural context.

## Study 1

**Figure 1.**
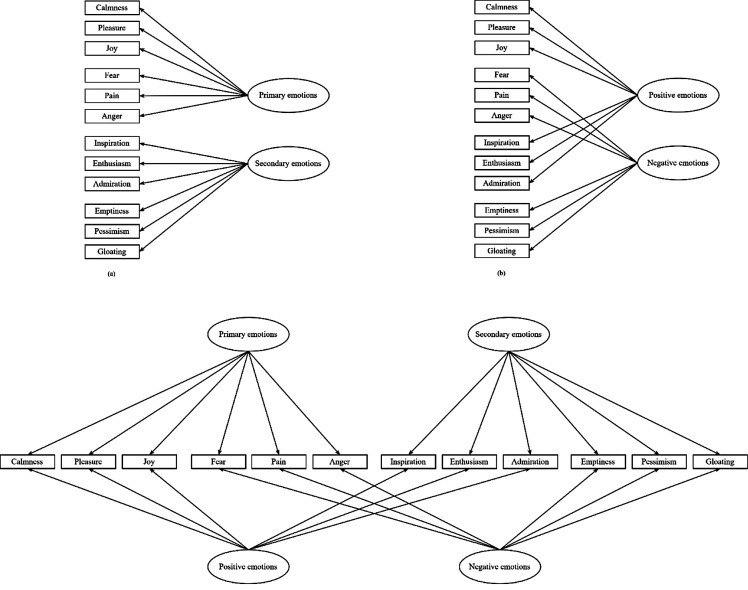
The theoretical models of emotions’ perception

The aim of this study was to test what role the valence and uniqueness of emotions play in their perception. We tested three alternative models of perceiving emotions (see *Figure 1*). According to infrahumanization theory, the uniqueness of emotions can be considered as a dominant feature in how those emotions are perceived, since secondary emotions are attributed regardless of their valence (Leyens et al. 2001; 2000). As a result, Model 1 suggests that in lay perception, the emotions selected in the preliminary study might be combined into two types according to their uniqueness and regardless of their valence: uniquely human and non-uniquely human emotions (Figure 1a).

At the same time, it has been repeatedly shown that the valence of information plays an important role in perception and attribution (Baumeister et al., 2001). Since we selected for analysis those emotions with different valence, we assume that in lay perception, they might be combined into two alternative types — positive and negative — based on their valence and regardless of their uniqueness, which is reflected in Model 2 (*Figure 1b*).

Finally, different information interacts in the process of perception, and the combination of various attributes of an object can lead to the emergence of unique patterns in perception. Such interactions can be observed between the emotional valence and emotional intensity (Mei et al., 2018). Each emotion we study has two attributes (valence and perceived uniqueness). Consequently, we can assume that both might be associated with how exactly this emotion will be perceived. Therefore, a third variant of the lay perception of the selected emotions is possible. Model 3 (bifactor) suggests that both attributes (uniqueness and valence) can interact in the perception, forming two dimensions (valence and uniqueness), so that each emotion simultaneously contributes to each of the dimensions (*Figure 1c*).

To check the proposed models, we used a confirmatory factor analysis (CFA) and the following fit indices to evaluate the fit of a model to the data: the ratio of chi-square to degrees of freedom, the root mean square error of approximation (RMSEA), the comparative fit index (CFI), the Tucker-Lewis index (TLI), and standardized root mean square residual (SRMR). According to Hu and Bentler (1999), values of .08 or below for RMSEA and .09 or below for SRMR indicate the model to be a good fit to the data. For CFI and TLI the values have to be at least .90 to indicate a good fit of a model (Kline, 2011). We also used the robust maximum likelihood (MLR) method since it robust to occurrences of data non-normality (Maydeu-Olivares, 2017). All analyses were made in the software Mplus (Muthen & Muthen, 2013).

## Method

### Participants

To control the quality of the data, attentiveness questions were used: 49 out of 292 respondents did not pass the attentiveness test and were excluded from the sample. As a result, the final sample included 243 respondents (51.0% women, *M_age_ =* 37.2*, SD =* 11.2), which were recruited for an online study through Yandex.Toloka (the Russian analog of Amazon’s Mechanical Turk) in return for a participation fee (0.7$). Most of the respondents (58.5%) had completed higher education, 29.3% had received secondary special education, and 12.3% were students. Fewer than a half of all respondents lived in cities with a population of more than a million (23.9% were from Moscow or Saint-Petersburg, and 15.2% from other large Russian cities); instead, most (54.3%) lived in cities with a population of fewer than a million people and 6.6% lived in villages.

### Procedure

Participants read an informed consent where they were informed that the study was voluntary and anonymous. Next, they answered questions on demographics (age, sex, education, and city of residence). After that, participants were offered a list of 12 emotions, where each emotion had to be rated on a 10-point scale of uniqueness (1 = *is not unique to humans: both humans and other living things are able to experience this emotion* to 10 = *is unique to humans: no one except humans are able to experience this emotion*) and valence (1 = *it is a negative emotion* to 10 = *it is a positive emotion*). On the last page, they were thanked for their participation.

## Results

### Descriptive analysis

*Table 1* presents the descriptive statistics for different emotions. Overall, positive emotions (M = 8.82, *SD =* 1.28) were rated higher on a valence item than negative emotions (M = 2.70, *SD* = 1.46), *Z* = –13.41, *p* < .001. On uniqueness item, the secondary emotions (M = 8.60, *SD* = 1.77) were rated higher (M = 3.57, *SD* = 2.11) than the primary ones, *Z* = –13.2, *p* < .001. These results confirm that emotions selected at the preliminary study differ in their perceived uniqueness and valence.

**Table 1:** The descriptive statistics for emotions

Emotions	Study 1								Study 2							
	Uniqueness				Valence				Uniqueness				Valence			
Pessimism	8.57	2.25	–1.67	1.95	2.77	1.96	1.13	0.78	7.52	2.73	–0.77	–0.59	2.77	2.05	1.12	0.69
gloating	8.74	2.34	–1.93	2.64	1.96	1.89	2.57	6.72	7.71	2.85	–0.99	–0.37	2.33	2.09	1.74	2.46
admiration	8.41	2.33	–1.49	1.25	8.56	1.76	–1.28	1.52	6.98	2.81	–0.75	–0.47	8.65	1.85	–1.52	1.79
emptiness	8.29	2.32	–1.43	1.21	2.78	2.25	1.49	1.65	6.96	2.78	–0.49	–0.88	2.75	2.01	1.33	1.57
enthusiasm	8.45	2.25	–1.59	1.84	9.01	1.66	–2.08	4.51	8.19	2.47	–0.73	–0.57	8.73	2.03	–1.40	1.50
inspiration	9.15	1.76	–2.34	4.97	9.16	1.53	–2.22	4.85	7.19	2.70	–1.37	0.97	8.60	1.79	–1.96	3.54
Pleasure	4.14	2.89	0.48	–1.04	8.67	1.80	–1.27	0.75	4.13	3.10	0.37	–1.26	8.73	1.86	–1.59	1.94
Joy	4.02	2.80	0.43	–1.09	9.27	1.63	–2.95	9.49	4.29	3.07	0.39	–1.19	9.12	1.74	–2.42	6.08
Fear	2.91	2.59	1.22	0.37	3.62	2.27	–0.80	0.26	3.23	2.81	0.94	–0.28	2.77	2.04	1.07	0.52
calmness	3.71	2.76	0.65	–0.75	8.24	2.03	–1.25	1.19	3.97	2.86	0.42	–1.02	8.31	1.97	–1.13	0.55
anger	3.97	2.90	0.59	–0.88	2.56	2.01	1.40	1.51	3.76	2.87	0.68	–0.76	2.63	2.17	1.35	1.17
Pain	2.69	2.44	1.35	0.83	2.51	2.03	1.45	1.69	3.24	2.81	0.91	–0.41	2.52	2.21	1.49	1.57

### Descriptive analysis

We tested Model 1 based on the uniqueness measure. The results of the CFAs are presented in *Table 2* and clearly show that Model 1 demonstrates a poor fit to the data. Adding to Model 1 the covariances between the estimates of emotions included in one type (admiration and inspiration; joy and anger; joy and fear) somewhat improves its fit, but still does not make it acceptable. This result means that in lay thinking, the uniqueness of emotions is not a main factor in the perception of emotions^2^.

**Table 2:** Closeness of fit indicators for different models

Models		χ^2^	*df*	RMSEA [90% CI]	SRMR	CFI	TLI	AIC
Model 1	Study 1	167.08***	53	.094 [.078; .110]	.085	.884	.856	12216
	Study 1 (adjusted model)	135.22***	50	.084 [.067; .101]	.082	.913	.886	12175
	Study 2	438.89***	53	.123 [.112; .134]	.105	.832	.790	25486
	Study 2 (adjusted model)	309.18***	49	.105 [.094; .116]	.100	.886	.847	25311
Model 2	Study 1	107.35***	53	.065 [.047; .083]	.054	.917	.896	11006
	Study 1 (adjusted model)	79.51**	51	.048 [.026; .068]	.053	.956	.943	10976
	Study 2	114.16***	53	.049 [.037; .061]	.031	.964	.955	21230
Model 3	Study 1	55.65*	38	.044 [.013; .067]	.025	.982	.969	12108
	Study 2	85.63***	40	.049 [.034; .063]	.026	.980	.967	25053

Note. df — degree of freedom; RMSEA — root mean square error of approximation; CFI — comparative fit index; TLI — Tucker Lewis index; SRMR — standardized root mean square residual; AIC — Akaike information criterion. *** —p < .001, ** —p < .01, * —p < .05.

Model 2 was tested based on its valence measure. The results of the CFA for Model 2 also demonstrates a poor fit to data. Adding to Model 2 the covariances between gloating and anger and between joy and enthusiasm makes the model a good fit. This point is important because covariances arise between negative emotions, one of which can be attributed to primary emotions, and another to secondary, indicating that valence can play a more significant role than uniqueness in emotions’ perception. The same situation occurs with the covariance between positive emotions.

Model 3 was also tested based on uniqueness measure^3^. In this model, we used two bases for grouping emotions: valence and perceived uniqueness (see Figure 1c). Table 2 shows that, in contrast to previous models, Model 3 fits the data well without adding additional covariances.

Overall, the results of this study show that although emotions are differentiated when they are rated high or low in their uniqueness, this rating is not related to how emotions are grouped in lay perception. In contrast, the perceived differences in the valence of emotions are significant when they are grouped.

It is equally important that if to the division of emotions based on valence we add the second attribute of emotions (in our case, the supposed division of emotions into primary and secondary), then the final model will describe the patterns of lay perception even better. In other words, both emotions’ valence and uniqueness can play an important role in their perception.

## Study 2

The main aim of this study was to replicate and check the reliability of Study 1 results regarding the role of valence and uniqueness of emotions in the context of the infrahumanization theory. We tested three alternative models of perceiving emotions as in the previous study. We expect confirmation of past conclusions about the role of valence and the uniqueness of emotions in their perception.

## Method

### Participants

Participants were recruited for an online study through Yandex.Toloka in return for a fee (0.7$). After checking for attentiveness, 128 out of 610 respondents were excluded due to poor quality of the data. The final sample included 482 participants (50.6 % women, *M_age_* = 34.89, *SD* = 11.32). Most respondents (57.7%) had completed higher education, 20.7% had received secondary special education, and 12.0% were students. Most of the participants (78.6%) noted their ethnicity as Russian, 4.7% as Ukrainians, the rest chose other ethnic groups. Fewer than half of the respondents lived in cities with a population of more than a million (17.4% were from Moscow or Saint-Petersburg, and 22.4% from other large Russian cities). Almost half of the respondents (47.7%) lived in cities with a population of fewer than a million people and 7.9% lived in villages.

### Procedure

The procedure and measures were the same as in Study 1.

## Results

To demonstrate the robustness of our results regarding emotions’ perception, we tested three models from Study 1 using data from Study 2. Descriptive statistics for emotions are presented in *Table 1*, and the results of the CFAs are presented in *Table 2*.

The results obtained completely repeated the patterns identified in Study 1. Model 1, describing the separation of emotions based on their perceived uniqueness, shows the worst fit to the data. Model 2, describing the separation of emotions based on valence, fits well with the data, but the best model is Model 3 (bifactor), which has better indicators than Model 2. These results once again confirm that in the lay perception both the valence and uniqueness of emotions play an important role^4^.

## Discussion

The goal of this study was to test the role of valence and uniqueness in the perception of emotions. Using the example of primary and secondary emotions of different valence specially selected for the Russian socio-cultural context, two studies were carried out. The studies’ results demonstrated the role of valence and its connection with the uniqueness of emotions. In general, the results obtained demonstrate several important points for understanding and measuring infrahumanization.

We found that emotions’ valence and uniqueness are important in the perception of these emotions. Other researchers have also highlighted the importance of emotions’ valence in the infrahumanization research but based on the attribution of emotions. Bain et al. (2009) highlighted the main effect of valence; Viki and Abrams (2003) confirmed the significance of emotions’ valence and interaction between emotions’ valence and uniqueness. Based on these results, they proposed that the valence effect can complement the infrahumanization effect in the attribution of emotions to the outgroups.

The repeated confirmation of the role of emotions’ valence in their attribution appears justified in the context of knowledge about the characteristics of perception and categorization. It has been repeatedly shown that people are guided by the most accessible feature in perception and attribution, and the valence of information is often just such a feature (Gräf & Unkelbach, 2016). Valence is an explicit feature that is simply accessible when perceiving emotions and can be culturally universal (for example, an emotion such as disgust has a negative valence in most cultures, while happiness has a positive one; An et al., 2017).

In contrast, the situation with the perceived uniqueness of emotions is much more complicated. Our two studies have shown that when respondents are explicitly asked to what extent a certain emotion is unique (experienced only by people) or non-unique (experienced by both people and other animals), they can differentiate emotions, but this does not lead to ‘uniqueness’ becoming the feature that groups emotions into different factors (as in the case of emotions’ valence). Eyssel and Ribas (2012) formulated a similar conclusion about the differentiation of emotions in their study.

As a result, the thesis of the infrahumanization theory – that the uniqueness of emotions dominates over their valence when attributing secondary emotions to the ingroup and outgroup – requires additional study since our conclusions about their perception do not confirm it.

In general, even though our findings generally repeat many of the results obtained previously in the framework of the infrahumanization theory, we propose looking at them from a new angle and carefully evaluating the role of valence in attribution of emotions. Such an assessment has important practical implications for research within the framework of the infrahumanization theory and the correct interpretation of the results obtained. Our results allow us to draw two important conclusions.

The first conclusion is that the valence of emotions is undoubtedly important in their perception and attribution, so controlling the valence effect should be an obligatory part of infrahumanization research. In addition, due to the accumulated findings on the role of valence in the attribution of emotions, some assumptions of the infrahumanization theory should be clarified for example, by clarifying the factors or conditions in which valence may not play a key role in attributing secondary emotions. Since several studies find confirmation of the infrahumanization effect, it would be productive to comprehend the results that do not find such confirmation.

The second conclusion is related to the understanding of the ‘uniqueness’ of emotions proposed by Leyens et al. (2001). As our results show, in lay perceptions emotions are not grouped on this basis; therefore, it cannot be concluded that uniqueness plays a role in the attribution of emotions. It is likely that some more significant grounds combine certain primary and secondary emotions into new groups, and it is these grounds that can be significant in attributing emotions. In other words, the interpretation of the results obtained in relation to the uniqueness of emotions precisely as a greater or lesser attribution of humanity to groups may raise doubts. Discussion of this problem could lead to the productive development of the infrahumanization theory.

## Limitations

This study meets limitation since we tested the models of emotions’ perception while the attribution of emotion also has a significant role for Infrahumanization theory. The additional analysis focused on the attribution of selected emotions to various social group can expand our understanding of the role of uniqueness and valence of emotions in interpersonal and intergroup relations.

## Data Availability

The dataset is freely available at Open Science Framework: https://osf.io/m2zaf/?view_only=dcb1827d08c1470ebf7441a9ef4d8797

## Footnotes

1 The full descriptive statistics for emotions can be find on OSF

2 Factor loadings for each emotion are presented on OSF (Study 1. Figures)

3 The use of the valence measure as the base for Model 3 does not change the results, and the bifactor model in this version also demonstrates good data fit.

4 Factor loadings for each emotion are presented on OSF (Study 2. Figures)
